# Parent-Reported Psychological Adjustment and Health-Related Quality of Life in Children with Growth Hormone Deficiency Before and After Six-Month Recombinant Growth Hormone Treatment, in Age-Matched Children with Familial Short Stature and in Normal-Statured Children

**DOI:** 10.3390/jcm15041394

**Published:** 2026-02-10

**Authors:** Anna Guerrini Usubini, Nicoletta Marazzi, Laura Abbruzzese, Gianluca Castelnuovo, Alessandro Sartorio

**Affiliations:** 1 Experimental Laboratory for Auxo-Endocrinological Research, Istituto Auxologico Italiano, Istituto di Ricovero e Cura a Carattere Scientifico (IRCCS), 28824 Piancavallo-Verbania, Italysartorio@auxologico.it (A.S.); 2 Division of Auxology, Istituto Auxologico Italiano, Istituto di Ricovero e Cura a Carattere Scientifico (IRCCS), 28824 Piancavallo-Verbania, Italy; 3 Psychology Research Laboratory, Istituto Auxologico Italiano, Istituto di Ricovero e Cura a Carattere Scientifico (IRCCS), 28824 Piancavallo-Verbania, Italy; 4 Department of Psychology, Catholic University, 20123 Milan, Italy

**Keywords:** growth hormone deficiency, short stature, quality of life, child behaviour checklist, quality of life in short stature youth

## Abstract

**Background:** Parental perceptions represent a crucial and underexplored dimension in evaluating the psychological adjustment and health-related quality of life (HRQoL) of children with growth hormone deficiency (GHD). This study aimed to: i. assess the psychological adjustment and HRQoL of children with GHD and to examine the psychological impact of six months of recombinant GH (rec-GH) therapy, based on parental reports; ii. compare the results obtained in parents of children with GHD with those of parents of children with familial short stature (FSS) and parents of children with normal stature (NS). **Methods:** Parents of 10 children with GHD, of 15 children with FSS, and of 17 children with NS completed the Child Behaviour Checklist for Children (CBCL) and the Quality of Life in Short Stature Youth (QoLISSY) questionnaires. For the GHD subgroup, assessments were repeated after six months of rec-GH treatment. **Results:** Parents reported a comparable overall behavioural functioning across the three subgroups, with a trend toward greater emotional difficulties in the GHD subgroup—particularly in the withdrawal/depression subscale of CBCL. After 6 months of rec-GH therapy, CBCL scores suggested a partial normalization of emotional functioning according to the parents of children with GHD. However, conduct problems remained more pronounced compared to the NS and FSS subgroups. As far as HRQoL is concerned, there were no significant differences between the parents of the GHD and FSS subgroups in the QoLISSY; however, both subgroups reported a markedly lower QoL than parents of children with NS across Physical, Social, Emotional, Future, Effect, and Total domains. Following a six-month rec-GH treatment, the GHD subgroup continued to exhibit lower physical QoL scores despite an improved height velocity, suggesting that the six-month rec-GH therapy did not fully mitigate the perceived physical limitations. **Conclusions:** This study provides an integrative evaluation of the psychosocial adjustment and HRQoL in children with GHD, FSS, and NS, emphasising the value of parent-reported outcomes.

## 1. Introduction

Growth hormone deficiency (GHD) is an endocrine disorder characterised by an insufficient secretion of the growth hormone (GH) from the anterior pituitary gland, leading to an impaired somatic growth and altered metabolic homeostasis [1,2,3,4]. In children, GHD primarily manifests as a short stature and delayed skeletal maturation, whereas in adults it is associated with a reduced lean body mass, increased adiposity, dyslipidemia, and diminished quality of life (QoL). The aetiology of GHD can be congenital, genetic, or acquired; however, in most cases, its aetiology is unknown and is referred to as idiopathic GHD [5]. GH replacement therapy represents the standard treatment for patients with GHD, aimed at enhancing growth velocity to support the achievement of normal stature during childhood and ultimately an adult height consistent with the child’s genetic potential [6].

Along with their physical growth impairment, a short stature associated with GHD is associated with severe psychosocial challenges which include anxiety, depression, and cognitive impairment [7], as well as behavioural and emotional problems [8,9]. Compared with their peers, children with GHD are reported to have higher rates of behavioural problems, emotional difficulties, and low self-esteem [10,11], all of which contribute to a diminished HRQoL [12]. The impact of growth hormone deficiency (GHD) on children’s quality of life is typically assessed through both children’s and parents’ self-reports. However, discrepancies between child- and parent-reported outcomes often emerge, reflecting differences in perspectives, experiences, and perceived burden.

Several studies have consistently shown that parents report a greater psychosocial and treatment burden associated with short stature and its management than children themselves. Specifically, parents tend to rate their child’s quality of life lower than children do, particularly in domains related to physical and psychosocial functioning before GH treatment [13,14].

According to Quitmann and colleagues [10], discrepancies between parent- and child-reported quality of life may arise from multiple factors, such as the quality of parent–child relationships and the social support perceived by the child. These discrepancies also vary depending on whether generic or condition-specific quality-of-life measures (e.g., QoLISSY) are used.

Including parent-reported outcomes is essential for a comprehensive understanding of the well-being of children and adolescents with GHD. Parents are typically the primary observers of their children’s everyday behaviour, emotional functioning, and treatment burden, providing a complementary perspective on their child’s physical, emotional, and social condition [13,14]. This is especially relevant when children are young, have cognitive or emotional impairments, or when self-reporting may be less reliable [14]. Moreover, parental perceptions of their child’s well-being and quality of life can influence healthcare utilization, treatment decisions, and adherence [15].

Despite a growing recognition of the importance of parental reports, gaps remain in understanding how short-term GH therapy impacts child well-being from the parent’s perspective, and how these outcomes compare across different groups of children. To address these gaps, the study was designed to: i. assess the psychological adjustment and HRQoL of children with GHD and to examine the psychological impact of six months of recombinant GH (rec-GH) therapy based on parental reports; ii. compare the results obtained in parents of children with GHD with those of parents of children with familial short stature (FSS) and of parents of children with normal stature (NS).

## 2. Materials and Methods

### 2.1. Participants and Procedures

The study involved parents of three subgroups of children divided as follows, according to the Italian reference growth charts for age and sex: subgroup 1, composed of 17 children with normal stature (NS); subgroup 2, consisting of 15 children with familial short stature (FSS); subgroup 3, composed of 10 children with GH deficiency (GHD). Only one parent per child was included in each subgroup.

Children with FSS and GHD were consecutively recruited at the Research Center for Growth Disorders, Istituto Auxologico Italiano, IRCCS, Milan, Italy. In contrast, children of the normal stature subgroup (NS) were recruited from the sons/daughters of the hospital’s medical, research, and administrative staff, as well as from the sons/daughters of friends and colleagues. Children with FSS were characterised by short stature, observed in other family members (not necessarily the parents), height within the expected range for the parental target height, a proportionate physical appearance without significant clinical signs, the expected timing of pubertal development, and bone age corresponding to the chronological age. Children with GHD were characterised by short stature (height standard deviation score, SDS ≤ −3 or height SDS ≤ −2 with a growth velocity per year of ≤−1.0 SDS for age and sex evaluated over at least 6 months) and GH peak levels of <8 ng/mL during two different pharmacological stimulation tests. A brain magnetic resonance imaging (MRI) scan was performed to exclude the presence of pathological conditions.

Parents were included if they were the parents of children with FSS, GHD, or NS aged 6 to 13 years; were Italian speaking; and provided informed consent. Parents were excluded if they had any form of physical or psychiatric impairment that could interfere with their participation in the study, or if they did not provide informed consent. Informed consent was obtained from both parents and children.

Participation in the study was voluntary, and no compensation was provided to the participants.

Once enrolled, parents were asked to provide socio-demographic data and complete two self-report questionnaires under the supervision of trained psychologists, as described below. The parents of the GHD subgroup were assessed twice—at baseline (before children started GH therapy) and after six months of rec-GH treatment (at a daily dose of 0.025–0.035 mg/kg body weight or 0.7–1.0 mg/m^2^ body area), corresponding to the visit for the renewal of the therapeutic plan. The parents of the FSS and NS subgroups completed the same assessments once at baseline. Questionnaires were administered in a quiet, standardised environment, in the presence of a clinical psychologist.

Recruitment took place between July 2023 and July 2025.

The study was approved by the Ethical Committee (EC) of Istituto Auxologico Italiano, IRCCS, Milan, Italy (approval number EC: 2023_03_21_03; research code: 01C312; acronym: PSICOSHORT). The research was conducted in accordance with the Declaration of Helsinki and its subsequent revisions.

### 2.2. Measures

Parents’ perceptions of emotional and behavioural problems in children were assessed using the Italian version of the Child Behaviour Checklist for Ages 6–18 (CBCL/6–18) [16,17]. The CBCL is a standardised, parent-report instrument within the Achenbach System of Empirically Based Assessment (ASEBA). It is widely used to evaluate a broad range of emotional and behavioural problems in children and adolescents aged 6 to 18 years. The CBCL/6–18 comprises 113 items that describe specific child behaviours, each rated by parents on a 3-point Likert scale ranging from 0 (“Not true”) to 2 (“Very true or often true”) according to the child’s behaviour during the preceding six months. The items provide eight empirically derived syndrome scales—Anxious/Depressed, Withdrawn/Depressed, Somatic Complaints, Social Problems, Thought Problems, Attention Problems, Rule-Breaking Behaviour, and Aggressive Behaviour. These syndrome scales can be summarised into two broad-band dimensions: Internalising Problems (reflecting anxiety, depression, and somatic complaints) and Externalising Problems (reflecting rule-breaking and aggressive behaviours). In addition, the CBCL provides six DSM-oriented scales aligned with the diagnostic categories of the Diagnostic and Statistical Manual of Mental Disorders (DSM): Affective Problems, Anxiety Problems, Somatic Problems, Attention Deficit/Hyperactivity Problems, Oppositional Defiant Problems, and Conduct Problems. Higher scores on these scales indicate greater levels of emotional or behavioural difficulties as perceived by the parent. The CBCL has demonstrated robust psychometric properties, including a high internal consistency, test–retest reliability, and cross-cultural validity, supporting its widespread use in both clinical and research contexts. In our sample, the internal consistency of the subscales ranged from 0.433 (Affective Problems) to 0.767 (Anxiety Problems). The only subscale with a low internal consistency was the Thought Problems subscale (α = 0.112).

Parents’ perceptions of HRQoL were assessed using the Quality of Life in Short Stature Youth (QoLISSY) questionnaire—Parent Version [18]. The QoLISSY is a disease-specific instrument developed to measure QoL in children and adolescents with short stature due to growth hormone deficiency (GHD) or idiopathic short stature (ISS). The Parent Version of the QoLISSY assesses parents’ perceptions of their child’s well-being and the psychosocial impact of short stature. The questionnaire consists of 46 items, organised into core and optional modules covering the following domains: Physical (e.g., physical functioning, energy); Emotional (e.g., self-esteem, mood); Social (e.g., peer relationships, social functioning); Coping and Beliefs about treatment and treatment burden (optional modules); Future (expectations and concerns regarding the child’s future); and Effect (perceived impact of the child’s short stature on family functioning and parental emotions). Items are rated on a 5-point Likert scale, from 1 (“strongly disagree”) to 5 (“strongly agree”), with higher scores reflecting a better perceived QoL. Scale scores can be transformed to a 0–100 metric to facilitate interpretation and comparison across domains. The QoLISSY Parent Version has demonstrated a high internal consistency, test–retest reliability, and construct validity, supporting its use in both clinical trials and observational studies assessing the psychosocial outcomes of growth-related disorders. It was initially created within a cross-cultural European project and has been translated and validated into several languages, including Italian, demonstrating good psychometric properties and conceptual equivalence across versions. In our sample, the internal consistency of the subscales ranged from 0.818 (Physical subscale) to 0.924 (Social subscale). For all licencing and permission requests for QoLISSY, please contact IQVIA at **IQVIA_COAs@iqvia.com**. The use of the licenced materials in the form provided by IQVIA is allowed only in connection with the current project (“PSICOSHORT”), developed by Istituto Auxologico Italiano, IRCCS, Milan, Italy. The questionnaire was administered only by the principal investigator (A.S.) or his coworkers to patients/controls participating in the project.

### 2.3. Statistical Analysis

Descriptive statistics were computed for all socio-demographic, clinical, and psychological variables. Measures of central tendency (mean, median) and dispersion (standard deviation, interquartile range) were reported, as appropriate for the distribution of the data. Given the relatively small sample size, non-parametric statistical procedures were applied throughout the analyses.

Between-subgroup comparisons were performed using the Kruskal–Wallis one-way analysis of variance (ANOVA) to detect differences in QoLISSY and CBCL scores among the three study subgroups: NS, FSS, and GHD.

When the Kruskal–Wallis test indicated significant subgroup differences, pairwise post hoc comparisons were conducted using Dunn’s test with the Bonferroni correction to control for multiple comparisons.

Pre- to post-treatment differences in QoLISSY and CBCL scores within the GHD subgroup were examined using the Wilcoxon signed-rank test.

Finally, to further investigate whether post-treatment outcomes in the GHD subgroup would become quite similar to the psychological profiles of the SS and NS subgroups, an additional non-parametric ANOVA was conducted comparing GHD post-treatment, FSS, and NS scores on the QoLISSY and CBCL scales. This analysis allowed for a direct evaluation of treatment-related convergence in QoL and behavioural adjustment across subgroups.

All analyses were performed using Jamovi statistical software (version 2.6.26). Statistical significance was set at *p* < 0.05. Effect sizes (ε^2^) were also calculated, where applicable, to provide an estimate of the magnitude of observed effects.

## 3. Results

### 3.1. Sample Characteristics (Parents and Children)

The study sample consisted of parents of three subgroups of children: in subgroup 1 (NS children), 17 parents completed the questionnaires (12 mothers and 5 fathers; the mean height SDS ± SD was −1.86 ± 0.62 for mothers and −1.06 ± 1.40 for fathers). Fourteen mothers (82.35%) were employed, one (5.88%) was self-employed, and two (11.76%) were unemployed. Among fathers, 11 (64.71%) were employed, and 6 (35.29%) were self-employed. In subgroup 2 (FSS children), 15 parents completed the questionnaires (10 mothers and 5 fathers; the mean height SDS ± SD was −0.21 ± 0.38 for mothers and −0.27 ± 0.99 for fathers). Twelve mothers (80%) were employed, and three (20%) were self-employed. Among fathers, nine (60%) were employed, five (33.33%) were self-employed, and one (6.67%) was unemployed. In subgroup 3 (GHD children), 10 parents completed the questionnaires (6 mothers and 4 fathers; the mean height SDS ± SD was −0.20 ± 1.17 for mothers and −0.64 ± 0.94 for fathers). Six mothers (60%) were employed, two (20%) were self-employed, and two (20%) were unemployed. Among fathers, six (60%) were employed, and four (40%) were self-employed.

Demographic characteristics of children are presented in Table S1.

### 3.2. Comparisons Between NS, FSS and GHD in CBCL and QoLISSY

A non-parametric one-way ANOVA (Kruskal–Wallis test) was conducted to compare CBCL subscale scores across the three subgroups (NS vs. FSS vs. GHD).

The results revealed no statistically significant subgroup differences across most CBCL scales (all *p* > 0.05), though some differences became quite significant. In particular, the CBCL Affective Problems scale showed a marginally significant difference among subgroups (χ^2^(2) = 5.982, *p* = 0.050, ε^2^ = 0.146), suggesting a moderate effect size. Similarly, CBCL Withdrawn/Depressed (χ^2^(2) = 5.510, *p* = 0.064, ε^2^ = 0.134) and CBCL Social Problems (χ^2^(2) = 5.138, *p* = 0.077, ε^2^ = 0.125) showed trends toward significance.

To further explore these trends, post hoc pairwise comparisons were conducted using the Dwass–Steel–Critchlow–Fligner (DSCF) method. The only pairwise comparison that reached statistical significance was between the GHD and NS subgroups on the CBCL Withdrawn/Depressed scale (W = −3.6069, *p* = 0.029), indicating that, according to their parents, children with GHD exhibited higher withdrawn/depressive symptoms than those in the NS subgroup.

All other pairwise contrasts were non-significant (*p* > 0.05). The results are presented in Table S2.

A non-parametric one-way ANOVA (Kruskal–Wallis test) was also conducted to compare the QoLISSY subscale scores across the three subgroups (NS vs. FSS vs. GHD). The results are presented in Table S3.

Significant subgroup effects were found for almost all domains except Coping and Beliefs. Specifically, the Kruskal–Wallis test indicated significant differences among the three subgroups for Physical (χ^2^ = 27.11, *p* < 0.001, ε^2^ = 0.66), Social (χ^2^ = 30.16, *p* < 0.001, ε^2^ = 0.74), Emotional (χ^2^ = 19.03, *p* < 0.001, ε^2^ = 0.46), Future (χ^2^ = 19.91, *p* < 0.001, ε^2^ = 0.49), Effect (χ^2^ = 16.55, *p* < 0.001, ε^2^ = 0.40), and Total QoLISSY scores (χ^2^ = 28.61, *p* < 0.001, ε^2^ = 0.70). Large effect sizes were observed for all QoLISSY domains related to physical, social, emotional functioning, future concerns, and overall quality of life (ε^2^ ranging from 0.40 to 0.74), indicating substantial subgroup differences. In contrast, the Coping and Beliefs domains showed only small effect sizes (ε^2^ = 0.0778 and 0.0636), suggesting minimal subgroup-related variation in these areas.

Post hoc pairwise comparisons revealed that, across the majority of domains showing global significance, the same pattern emerged: parents’ reports for the GHD subgroup did not differ significantly from those for the FSS subgroup (all *p* > 0.05). Parents of children in both the GHD and FSS subgroups reported significantly lower QoL scores compared with those of children with NS across the Physical, Social, Emotional, Future, Effect, and Total QoLISSY domains (all *p* ≤ 0.002).

Medians and interquartile ranges for all the CBCL and QoLISSY subscales across the three subgroups are presented in Table S4.

### 3.3. Pre- to Post-Treatment Differences in the CBCL Scores of Parents of the GHD Subgroup

A non-parametric Wilcoxon signed-rank test was conducted to compare CBCL and QoLISSY scores in parents of children with growth hormone deficiency (GHD) at baseline (before children started GH therapy) and after six months of rec-GH treatment.

As far as CBCL scores are concerned, significant pre- to post-treatment differences were observed in the Affective Problems (*p* = 0.008), Withdrawn/Depressed (*p* = 0.004), Social Problems (*p* = 0.030), Other Problems (*p* = 0.021), and Total Problems (*p* = 0.032) scales, with lower scores at post-treatment compared with pre-treatment. No other significant pre- to post-treatment differences were found in the CBCL scores of parents in the GHD subgroup. The results are presented in Table S5.

As for QoLISSY scores, the results showed non-significant pre- to post-treatment differences in all QoLISSY subscales, except for the Emotional subscale (*p* = 0.020), with lower post-treatment scores than pre-treatment. The results are presented in Table S6.

### 3.4. Comparisons Between Parents’ Reports on CBCL and QoLISSY of GHD (Post-Treatment), FSS, and NS Subgroups

To further investigate whether post-treatment outcomes in the GHD subgroup would become quite similar to the psychological profiles of the FSS and NS subgroups, an additional non-parametric ANOVA was conducted comparing GHD post-treatment, FSS, and NS scores on the CBCL scales.

Few significant subgroup differences were found across the CBCL subscales. The only statistically significant effect was found for Conduct Problems (χ^2^ = 8.21, *p* = 0.017, ε^2^ = 0.20), indicating a moderate effect size. A near-significant trend also emerged for Rule-Breaking Behaviour (χ^2^ = 8.80, *p* = 0.012) and Externalising Problems (χ^2^ = 5.66, *p* = 0.059), suggesting that behavioural regulation difficulties might distinguish subgroups to some extent.

Pairwise comparisons indicated that parents rated the GHD children at post-treatment as having more conduct and rule-breaking problems than the parents of NS children did (subgroup 1; *p* = 0.020 and *p* = 0.013, respectively). No significant differences were observed between the GHD and FSS subgroups (all *p* > 0.09), and the latter did not differ significantly from the parents’ reports of NS peers. Thus, even after GH therapy, parents continued to perceive GHD children as showing somewhat elevated externalising behaviours, particularly in Conduct and Rule-Breaking domains, relative to parents of NS children. No significant subgroup effects were detected for Internalising Problems (anxiety, affective, or somatic symptoms; all *p* > 0.08). This suggests that parental perceptions of emotional distress were broadly comparable across subgroups at the time of assessment, with no clear evidence of residual emotional difficulties in the GHD subgroup after treatment. Differences in Social Problems were significant (χ^2^ = 5.50, *p* = 0.064), with both GHD and FSS children tending to score higher than normal-height peers, though the difference did not reach statistical significance. No significant effects were detected for Attention or Thought Problems (all *p* > 0.50), suggesting that cognitive or attentional functioning was perceived similarly across subgroups. The Internalising, Externalising, and Total Problems scores showed no significant overall subgroup differences (all *p* > 0.05), although the near-significant externalising trend again reflected the elevated conduct and rule-breaking tendencies observed in children with GHD.

The results are presented in Table S7.

To further investigate whether post-treatment outcomes in the GHD subgroup would become quite similar to the psychological profiles of the FSS and NS subgroups, an additional non-parametric ANOVA was conducted comparing GHD post-treatment, FSS, and NS scores on the QoLISSY scales. The results showed that, in the Physical, Social and Emotional domains, both parents of GHD and FSS children scored significantly lower than parents of NS peers (all *p* < 0.001), but did not differ significantly from each other (*p* = 0.96; *p* = 0.93; *p* = 0.19, respectively).

This means that, despite being evaluated after treatment, the parents of GHD children perceived their child’s social and emotional QoL as comparable to that of untreated short children, and clearly lower than that of NS peers.

Similarly, for the Future (W = −5.06, *p* = 0.001) and Effect (W = −4.45, *p* = 0.005) domains, the parents of children with GHD reported significantly more concerns than the parents of NS children. However, the differences between GHD and FSS children were again non-significant (*p* = 0.73 and *p* = 0.93, respectively). No significant differences were found between subgroups in Coping or Beliefs (all *p* > 0.10), indicating that parents’ perceptions of their children’s coping strategies and personal beliefs were relatively stable and unaffected by diagnosis or treatment status. The overall QoLISSY Total score followed a consistent pattern: the parents of GHD children scored significantly lower than the parents of children with NS (W = −6.05, *p* < 0.001), but were not different from the parents of children with FSS (W = 1.65, *p* = 0.473).

The results are presented in Table S8. All tables are presented in the Supplementary Materials.

## 4. Discussion

The present study indicates that, according to their parents, children with GHD exhibit behavioural and quality-of-life profiles that are largely comparable to those of peers with normal stature, although difficulties in internalising behaviours, such as emotional withdrawal and depressive tendencies, may be more pronounced, consistent with previous findings [11,12,13,14,15,16,17,18,19]. Although the parents of children with GHD and FSS did not differ significantly in their reports regarding the QoL of their children, both subgroups reported markedly lower QoL scores than the parents of children with NS across Physical, Social, Emotional, Future, Effect, and Total scales. These results are broadly consistent with previous studies, highlighting the perceived impact of short stature on both physical and psychosocial functioning [15,16,17,18,19,20,21].

Six months of GH therapy were associated with improvements in internalising symptoms and social functioning, highlighting the early benefits of treatment on psychological adjustment. However, parent-reported quality of life, particularly in the emotional and physical domains, remained lower than that of children with normal stature, suggesting that GH therapy alone may not fully alleviate the psychosocial burden associated with short stature. Alternatively, the lack of improvements may be due to the short follow-up period relative to longer-term improvements reported in the literature [20].

By comparing the post-treatment GHD subgroup with the pre-treatment conditions of the other subgroups on the CBCL, the results showed a partial normalisation of emotional functioning in GHD children, as they did not differ significantly from FSS or NS peers on internalising CBCL domains. This is consistent with previous findings. Stabler et al. [22] observed that children with GHD demonstrated significant improvements in the CBCL internalising subscales after three years of growth hormone treatment. However, residual behavioural challenges, such as elevated conduct problems, were observed. Similarly, comparisons between the post-treatment GHD subgroup and the conditions of other subgroups for QoLISSY showed that the GHD subgroup continued to show the lowest physical QoL scores, suggesting that therapy did not fully recover the perceived physical limitations associated with short stature and that GH therapy alone may not fully resolve the psychosocial burden of short stature. These findings are consistent with previous reports showing that parent-perceived limitations may remain despite medical intervention [19].

The findings of the study underscore the importance of monitoring emotional well-being and providing psychosocial support alongside medical treatment, as parental perceptions and broader contextual factors continue to influence the child’s experience of health-related quality of life. Overall, while GH therapy contributes positively to some aspects of psychological adjustment, comprehensive interventions may be necessary to address residual emotional and functional challenges in children with GHD.

This study provides a comprehensive examination of psychosocial and quality-of-life outcomes in children with GHD, FSS, and NS by integrating parent-reported behavioural measures (CBCL) with a disease-specific QoL instrument (QoLISSY). The inclusion of three clinical subgroups allowed a differentiation between the effects of GHD and short stature per se, while the assessment of children with GHD after GH therapy offered insight into residual psychosocial challenges despite treatment. Conducting the study in an Italian context represents an additional strength, as this population has been underrepresented in the literature.

However, several limitations should be acknowledged. The small sample sizes in each subgroup limit generalisability and statistical power, requiring the use of non-parametric analyses and increasing the risk of Type I and II errors; therefore, the findings should be interpreted with caution and considered exploratory. The predominance of participants from northern Italy restricts the sample representativeness, and the relatively low internal consistency of some scales may have reduced measurement precision. Moreover, potential confounding factors, such as socioeconomic status and gender differences, were not systematically controlled.

Although six months of rec-GH treatment did not fully resolve psychosocial vulnerabilities, this short-term finding does not exclude meaningful improvements with longer treatment durations. Future research should involve larger, multicentre samples and longitudinal designs, include structured clinical interviews (e.g., K-SADS), and systematically account for relevant confounding variables to better clarify the psychosocial effects of prolonged GH therapy.

## Data Availability

Raw data will be uploaded to www.zenodo.org immediately after the manuscript is accepted and will be available upon reasonable request from the authors, A.G.U. and A.S.

## Supplementary Materials

The following supporting information can be downloaded at: https://www.mdpi.com/article/10.3390/jcm15041394/s1, Table S1: Demographic characteristics of children of NS, FSS and GHD subgroups; Table S2: Kruskal–Wallis test to compare CBCL subscale scores across the three subgroups; Table S3: Kruskal–Wallis test to compare QoLISSY subscale scores across the three subgroups; Table S4: Medians and interquartile ranges for all the CBCL and QoLISSY subscales across the three subgroups; Table S5: Pre- to post-treatment differences in the CBCL scores of parents of the GHD subgroup; Table S6: Pre- to post-treatment differences in the QoLISSY scores of parents of the GHD subgroup; Table S7: Kruskal–Wallis test to compare CBCL subscale scores of GHD (post-treatment), NS and FSS subgroups; Table S8: Kruskal–Wallis test to compare QoLISSY subscale scores of GHD (post-treatment), NS and FSS subgroups.
