# Supplementary material for: Parent-Reported Psychological Adjustment and Health-Related Quality of Life in Children with Growth Hormone Deficiency Before and After Six-Month Recombinant Growth Hormone Treatment, in Age-Matched Children with Familial Short Stature and in Normal-Statured Children
Source: J Clin Med. 2026 Feb 10;15(4):1394. doi: 10.3390/jcm15041394 (PMC12941226; doi:10.3390/jcm15041394)
Supplement: Supplementary file 1 [file jcm-15-01394-s001.zip › jcm-4122603-supplementary.pdf]

Table S1. Demographic characteristics of children of NS, FSS and GHD subgroups.

	NS subgroup (N= 17)	FSS subgroup (N= 15)	GHD subgroup (N= 10)	
	N (%)	N (%)	N (%)	
Gender				
Females	7 (41.18%)	6 (40%)	6 (60%)	
Males	10 (58.82%)	9 (60%)	4 (40%)	
Education				
Elementary school	10 (58.82)	5 (33.34%)	5 (50%)	
Middle school	7 (41.18%)	10 (66.66%)	5 (50%)	
Region of origin				
Northern Italy	17 (100%)	12 (80%)	7 (70%)	
Central Italy	-	3 (20%)	-	
Southern Italy	-		3(30%)	
	M (SD)	M (SD)	M (SD)	p value
Age	11.3±2.06	11.39±1.85	10.93±2.34	0.870
Heigh SDS	0.19+0.37	-1.93+0.56	-2.44+0.28	

Note: NS: normal stature; FSS: familial short stature; GHD: Growth hormone deficiency

Table S2. Kruskal-Wallis test to compare CBCL subscale scores across the three subgroups

	χ^2	p	ε^2
CBCL_affective problems	5.982	0.050*	0.14591
CBCL_anxiety problems	5.015	0.081	0.12233
CBCL_somatic problems	0.479	0.787	0.01169
CBCL_ADHD	1.089	0.580	0.02656
CBCL_oppositional defiant problems	0.570	0.752	0.01390
CBCL_conduct problems	3.617	0.164	0.08821
CBCL_anxius/depressed	4.800	0.091	0.11706
CBCL_withdrawn/depressed	5.510	0.064	0.13439
CBCL_somatic syndrome	0.628	0.730	0.01532
CBCL_social problems	5.138	0.077	0.12532
CBCL_though problems	0.949	0.622	0.02316
CBCL_attention problems	2.394	0.302	0.05840
CBCL_rule breaking behaviors	3.555	0.169	0.08670
CBCL_aggressive behaviors	1.849	0.397	0.04511
CBCL_other problems	0.287	0.866	0.00717

	χ^2	p	ε^2
CBCL_internalization	4.598	0.100	0.11215
CBCL_externalization	1.143	0.565	0.02788
CBCL_other scale	1.939	0.379	0.04729
CBCL_TOTAL	0.848	0.654	0.02068

Note: CBCL: Child Behaviour Checklist for Children.

*: statistically significant

Table S3. Kruskal-Wallis test to compare QoLISSY subscale scores across the three subgroups

	χ^2	p	ε^2
QoLISSY_physical	27.11	< 0.001*	0.6613
QoLISSY_social	30.16	< 0.001*	0.7356
QoLISSY_emotional	19.03	< 0.001*	0.4642
QoLISSY_coping	3.19	0.203	0.0778
QoLISSY_beliefs	2.61	0.272	0.0636
QoLISSY total	28.61	< 0.001*	0.6978
QoLISSY_future	19.91	< 0.001*	0.4855
QoLISSY_effect	16.55	< 0.001*	0.4037

Note: QoLISSY: Quality of Life in Short Stature Youth

*: statistically significant

Table S4. Medians and interquartile ranges for all the CBCL and QoLISSY subscales across the three subgroups

	NS subgroup (N=17)		FSS subgroup (N=15)		GHD subgroup (N=10)			
					Pre-treatment		Post-treatment	
	MD	IQR	MD	IQR	MD	IQR	MD	IQR
CBCL_affective problems	1.00	1.50	1.00	2.00	3.00	2.75	1.00	1.50
CBCL_anxiety problems	1.00	2.50	2.00	2.00	2.50	2.00	1.50	2.75
CBCL_somatic problems	1.00	1.50	0.00	1.00	1.00	1.75	1.00	1.00

CBCL_ADHD	2.00	2.50	1.00	2.00	2.00	1.00	2.50	2.75
CBCL_oppositional defiant problems	2.00	2.00	2.00	2.00	1.50	1.75	1.00	0.75
CBCL_conduct problems	1.00	1.50	0.00	4.00	0.50	1.75	0.00	0.00
CBCL_anxius/depressed	2.00	2.00	4.00	2.00	3.50	4.50	2.50	4.75
CBCL_withdrawn/depressed	1.00	1.50	1.00	2.00	3.00	1.75	1.50	2.00
CBCL_somatic sindrome	1.00	2.00	1.00	3.00	1.00	1.00	1.00	1.50
CBCL_social problems	1.00	1.00	2.00	1.00	3.00	2.50	1.00	1.75
CBCL_though problems	1.00	2.00	1.00	2.00	1.00	1.00	1.00	0.75
CBCL_attention problems	3.00	4.00	2.00	4.00	4.00	3.75	2.50	4.00
CBCL_rule breaking behaviors	2.00	2.00	1.00	4.00	0.50	1.00	0.00	0.00
CBCL_aggressive behaviors	3.00	3.50	4.00	3.00	2.00	3.00	1.50	1.75
CBCL_other problems	3.00	2.00	3.00	5.00	3.00	2.00	2.00	2.50
CBCL_internalization	4.00	3.00	7.00	6.00	8.50	5.00	6.50	9.75
CBCL_externalization	4.00	5.00	5.00	7.00	3.50	3.75	2.00	2.00
CBCL_other scale	9.00	6.50	8.00	11.00	11.50	4.25	8.00	2.00
CBCL_TOTAL	20.00	16.50	19.00	23.00	24.00	14.00	17.00	10.25
QoLISSY_physical	100.0	8.33	70.80	22.92	72.90	26.04	100.8	0.297
QoLISSY_social	100.0	0.00	56.30	35.94	76.60	29.69	70.30	32.81
QoLISSY_emotional	93.80	9.38	65.60	37.50	70.30	17.97	43.80	11.25
QoLISSY_coping	32.50	52.50	47.50	28.75	41.30	8.13	56.30	42.18
QoLISSY_beliefs	75.00	43.75	56.30	34.38	59.40	43.75	72.70	23.52
QoLISSY_total	96.90	2.78	69.80	30.03	77.40	23.00	77.10	11.45
QoLISSY_future	100.0	0.00	75.00	40.00	80.00	27.50	80.00	28.75
QoLISSY_effect	88.60	15.91	47.70	35.23	38.60	40.34	44.30	30.68

Note: CBCL: Child Behaviour Checklist for Children; QoLISSY: Quality of Life in Short Stature Youth.

Table S5. Pre- to post-treatment differences in the CBCL scores of parents of the GHD subgroup

Pre- to post-treatment	Wilcoxon W	p	Effect size r
CBCL_affective problems	45.0	0.008*	1.0000
CBCL_anxiety problems	25.0	0.359	0.3889
CBCL_somatic problems	11.5	0.915	0.0952
CBCL_ADHD	26.5	0.672	0.1778
CBCL_oppositional defiant problems	28.0	0.152	0.5556
CBCL_conduct problems	10.0	0.098	1.0000
CBCL_anxius/depressed	33.5	0.211	0.4889
CBCL_withdrawn/depressed	26.5	0.040*	0.8929
CBCL_somatic syndrome	18.0	0.551	0.2857
CBCL_social problems	49.0	0.030*	0.7818
CBCL_though problems	17.5	0.588	0.2500
CBCL_attention problems	26.0	0.285	0.4444
CBCL_rule breaking behaviors	10.0	0.095	1.0000
CBCL_aggressive behaviors	37.5	0.329	0.3636
CBCL_other problems	22.0	0.621	0.2222
CBCL_internalization	41.5	0.168	0.5091
CBCL_externalization	41.5	0.166	0.5091
CBCL_other scale	50.5	0.021*	0.8364
CBCL_TOTAL	49.0	0.032*	0.7818

Note: CBCL: Child Behaviour Checklist for Children

*: statistically significant

Table S6. Pre- to post-treatment differences in the QoLISSY scores of parents of the GHD subgroup

Pre- to post-treatment	Wilcoxon w	p	Effect size r
QoLISSY_physical	42.5	0.139	0.5455
QoLISSY_social	26.0	0.719	0.1556
QoLISSY_emotional	50.0	0.020*	0.8182
QoLISSY_coping	15.5	0.241	-0.4364
QoLISSY_beliefs	22.0	0.625	-0.2000
QoLISSY_total	21.5	0.575	-0.2182
QoLISSY_future	22.0	1.000	-0.0222
QoLISSY_effect	26.0	0.922	-0.0545

Note: QoLISSY: Quality of Life in Short Stature Youth.

*: statistically significant

Table S7. Kruskal-Wallis test to compare CBCL subscale scores of GHD (post-treatment), NS and FSS subgroups

	χ^2	p	ε^2
CBCL_affective problems	0.0340	0.983	8.30e-4
CBCL_anxiety problems	4.9262	0.085	0.12015
CBCL_somatic problems	0.2544	0.881	0.00620
CBCL_ADHD	0.5487	0.760	0.01338
CBCL_oppositional defiant problems	1.9355	0.380	0.04721
CBCL_conduct problems	8.2052	0.017*	0.20013
CBCL_anxius/depressed	3.4944	0.174	0.08523
CBCL_withdrawn/depressed	0.2259	0.893	0.00551
CBCL_somatic sindrome	0.0564	0.972	0.00138

	χ^2	p	ε^2
CBCL_social problems	5.4976	0.064	0.13409
CBCL_though problems	0.3883	0.824	0.00947
CBCL_attention problems	1.1843	0.553	0.02889
CBCL_rule breaking behaviors	8.8042	0.012*	0.21474
CBCL_aggressive behaviors	4.9946	0.082	0.12182
CBCL_other problems	1.2375	0.539	0.03018
CBCL_internalization	1.0204	0.600	0.02489
CBCL_externalization	5.6574	0.059	0.13799
CBCL_other scale	0.1637	0.921	0.00399
CBCL_TOTAL	0.8647	0.649	0.02109

Note: CBCL: Child Behaviour Checklist for Children.

*: statistically significant

Table S8. Kruskal-Wallis test to compare QoLISSY subscale scores of GHD (post-treatment), NS and FSS subgroups

	χ^2	p	ε^2
QoLISSY_physical	28.21	< 0.001*	0.6881
QoLISSY_social	29.79	< 0.001*	0.7267
QoLISSY_emotional	24.42	< 0.001*	0.5957
QoLISSY_coping	5.40	0.067	0.1318
QoLISSY_beliefs	2.53	0.283	0.0616
QoLISSY_total	29.01	< 0.001*	0.7077

	χ^2	p	ε^2
QoLISSY_future	18.86	< 0.001*	0.4600
QoLISSY_effect	15.29	< 0.001*	0.3730

Note: QoLISSY: Quality of Life in Short Stature Youth.

*: statistically significant
